# Down-Regulation of Vitamin D Receptor in Mammospheres: Implications for Vitamin D Resistance in Breast Cancer and Potential for Combination Therapy

**DOI:** 10.1371/journal.pone.0053287

**Published:** 2013-01-14

**Authors:** Shehla Pervin, Martin Hewison, Melissa Braga, Lac Tran, Rene Chun, Amer Karam, Gautam Chaudhuri, Keith Norris, Rajan Singh

**Affiliations:** 1 Department of Internal Medicine, Charles Drew University of Medicine and Science, Los Angeles, California, United States of America; 2 Department of Obstetrics and Gynecology, David Geffen School of Medicine at UCLA, Los Angeles, California, United States of America; 3 Department of Orthopedic Surgery, David Geffen School of Medicine at UCLA, Los Angeles, California, United States of America; 4 Jonsson Comprehensive Cancer Center at UCLA, David Geffen School of Medicine at UCLA, Los Angeles, California, United States of America; 5 Department of Molecular and Medical Pharmacology, David Geffen School of Medicine at UCLA, Los Angeles, California, United States of America; Seoul National University, Korea, Republic of

## Abstract

Vitamin D signaling in mammary cancer stem cells (MCSCs), which are implicated in the initiation and progression of breast cancer, is poorly understood. In this study, we examined vitamin D signaling in mammospheres which are enriched in MCSCs from established breast cancer cell lines. Breast cancer cells positive for aldehyde dehydrogenase (ALDH^+^) had increased ability to form mammospheres compared to ALDH^−^ cells. These mammospheres expressed MCSC-specific markers and generated transplantable xenografts in nude mice. Vitamin D receptor (VDR) was significantly down-regulated in mammospheres, as well as in ALDH^+^ breast cancer cells. TN aggressive human breast tumors as well as transplantable xenografts obtained from SKBR3 expressed significantly lower levels of VDR but higher levels of CD44 expression. Snail was up-regulated in mammospheres isolated from breast cancer cells. Inhibition of VDR expression by siRNA led to a significant change in key EMT-specific transcription factors and increased the ability of these cells to form mammospheres. On the other hand, over-expression of VDR led to a down-regulation of Snail but increased expression of E-cad and significantly compromised the ability of cells to form mammospheres. Mammospheres were relatively insensitive to treatment with 1,25-dihydroxyvitamin D (1,25D), the active form of vitamin D, compared to more differentiated cancer cells grown in presence of serum. Treatment of H-Ras transformed HMLE^HRas^ cells with DETA NONOate, a nitric oxide (NO)-donor led to induction of MAP-kinase phosphatase -1 (MKP-1) and dephosphorylation of ERK1/2 in the mammospheres. Combined treatment of these cells with 1,25D and a low-concentration of DETA NONOate led to a significant decrease in the overall size of mammospheres and reduced tumor volume in nude mice. Our findings therefore, suggest that combination therapy using 1,25D with drugs specifically targeting key survival pathways in MCSCs warrant testing in prospective clinical trial for treatment of aggressive breast cancer.

## Introduction

Compelling evidence has emerged that normal vitamin D levels are associated with reduced rates of various types of cancer, including breast tumors, in addition to its various other important functions [1]. Vitamin D insufficiency has been found in 76% of women with breast cancer and higher levels of vitamin D intake have been found to be associated with reduced risk and improved prognosis in breast cancer patients [2]. Vitamin D receptor (VDR) gene polymorphisms have also been shown to modify breast cancer susceptibility [3]–[5]. However, breast cancer cells that express functional estrogen receptor (ER^+^) also have high expression of VDR and display enhanced sensitivity to treatment with the active form of vitamin D (1,25-dihydroxyvitamin D, 1,25D) [6]–[7]. Poorly differentiated (ER^−^) breast cancer cell lines express less VDR and are less sensitive to 1,25D administration [7].

High numbers of mammary cancer stem cells (MCSCs) are found in higher grade aggressive and undifferentiated breast tumors compared to the differentiated lower grade tumors [8]–[10]. MCSCs also express embryonic stem (ES) cell-specific gene sets and arise from mammary stem cells (MSCs), where self-renewal/differentiation mechanisms are deregulated [11]. In addition to driving aggressive tumors, MCSCs have also been implicated in recurrence of less aggressive ER^+^ breast tumors following treatment [12]–[13]. Although primary tumors usually respond well to various therapeutic regimens, including 1,25D treatment, recurrent tumors are more aggressive and resistant [14]. The sensitivity of MCSCs to 1,25D treatment remains poorly understood and is the focus of this report.

In the current study, we used *in vitro* and *in vivo* approaches to examine the molecular mechanisms that regulate 1,25D sensitivity in mammospheres isolated from breast cancer cell lines. Our results indicate that mammospheres isolated selectively from established breast cancer cells have suppressed VDR signaling, increased expression of CD44 [15], and decreased sensitivity to 1,25D administration. Incubation with low concentrations (300 µM) of the nitric oxide (NO)-donor DETA NONOate) (which induces MAP-kinase phosphatase-1 and dephosphorylates ERK1/2 increased the sensitivity of mammospheres to 1,25D administration *in vitro*, and significantly reduced tumor volume in nude mice *in vivo*. Our data therefore, provides evidence that vitamin D or its analogs may be more effective as an adjuvant therapy, when used in combination therapy with other compounds known to effectively target key survival pathways in MCSCs.

## Materials and Methods

### Reagents and Cells

Basic human recombinant fibroblast growth factor (FGF) and recombinant human epidermal growth factor (EGF) were purchased from Calbiochem (San Diego, CA). 1,25(OH)_2_ D3 (1,25D) was obtained from Enzo Life Science (Plymouth, PA). Insulin, cholera toxin, and hydrocortisone were purchased from Sigma chemicals. DETA-NONOate was from Cayman Biochemical (Ann Arbor, Michigan). Human breast cancer cell lines MCF-7 and SKBR3 were obtained from ATCC (Rockville, MD). SKBR3 cells were cultured in RPMI 1640 (Sigma, St. Louis, MO) supplemented with L-glutamine and 10% fetal bovine serum (FBS). MCF-7 cells were cultured in DMEM containing sodium pyruvate, 10 mM non-essential amino acids, 2 mM L-glutamine, 10 g/ml insulin, and 10% FBS. HMLE and HMLE^H-RAS^ were obtained from Robert Weinberg (Whitehead Institute at MIT, Cambridge, Massachusetts). HMLE and HMLE^H-RAS^ (HRas) were cultured in DMEM: Ham’s F-12 (1∶1) supplemented with 10% FBS, 10 mM HEPES, 10 µg/ml insulin, 20 ng/ml EGF, 100 ng/ml cholera enterotoxin, and 0.5 µg/ml hydrocortisone.

### Mammosphere Formation

Mammosphere growth assays were carried out as described [16]. Cell lines were trypsinized and suspended in serum-free mammosphere media (MSM) comprised of DMEM: Ham’s F-12 (1∶1) supplemented with 10 mM HEPES, 5 µg/ml insulin, 20 ng/ml EGF and 10 ng/ml basic FGF. Cells were counted and 50,000 cells/well were plated in ultra-low attachment 6-well plates (Costar™ 3471).

### Immunofluorescence

Cells were fixed and prepared for immunofluorescence as previously described [17]. Primary antisera were used at 0.5–1 ug/ml. CD44 (10432), and CD24 (10424) were purchased from Stem Cell Technologies. ESA (E1591) was purchased from Ventana (Freemont, CA). MKP-1 and pERK1/2 antibodies were obtained from Santa Cruz Biotech (Santa Cruz, CA) and Cell Signaling (Danvers, MA) respectively. Secondary antisera included highly cross adsorbed goat anti-rabbit Alexa-633, goat anti-rabbit Alexa-488 or goat anti-mouse Alexa-488 (A31619) were obtained from Life Technology (Carlsbad, CA). Cells were counterstained with 6-diamidino-2-phenylindole (DAPI, Molecular Probes) and data were analyzed using Openlab 5.0 software (Improvision) [17].

### Aldefluor Assay and Flow Cytometry

To measure and isolate cells with high ALDH activity, the Aldefluor assay was carried out [16] according to manufacturer’s (Stem cell Technologies) guidelines. Briefly, breast cancer cells were suspended in Aldefluor assay buffer containing an ALDH substrate, bodipy-aminoacetaldehyde (BAAA) at 1.5 µM, and incubated for 40 min at 37°C. To distinguish between ALDH^+^ and ALDH^−^ cells, a fraction of cells was incubated under identical condition in the presence of a 10-fold molar excess of the ALDH inhibitor, diethyl amino benzaldehyde (DEAB). This results in a significant decrease in the fluorescent intensity of ALDH^+^ cells and was used to compensate the flow cytometer.

### PCR Array Analysis

Aliquots of total cellular RNA isolated with Trizol reagent from SKBR3 and HRas cells grown under plastic and mammospheres conditions were subjected to RT2 profiler PCR Array [Human Epithelial to Mesenchymal Transition (EMT), PAHS-090A; SA Bioscience, Frederick, MD] analysis. This PCR Array contains pre-dispensed primer sets for the specified genes into a 96-well PCR plate designed to study the expression profiles of 84 genes related to EMT. Raw data were analyzed using PCR Array Data Analysis, and fold changes in relative gene expression were presented after background correction and normalization with a house-keeping gene [18] using ΔΔ*C*_t_ method following the manufacturer’s instruction (SA Bioscience, Frederick, MD).

### Quantitative mRNA expression by Real time PCR

Total RNA was extracted from cultured cells using Trizol method. cDNAs were synthesized from 1 µg total RNA using GeneAmp RNA PCR core kit (Applied Bio systems N808-0143) and 10 times diluted cDNA was used for PCR template. Human PCR primers were designed and obtained from Primer Bank DNA Core facility (http://pga.mph.harvard.edu/primerbank, MGH Harvard, Cambridge, MA). The following primer sets were used - c-Myc (139 bp), 306–328/444–424 on AF534403; Klf-4 (54 bp), 13–31/66–47 on NM_004235; Oct-4 (78 bp), 157–175/234–214 on NM_001159542; Sox-2 (200 bp), 121–140/320–301 on NM_006943; VDR (60 bp), 605–627/664–644 on NM_000376; RXR (69 bp), 45–65/113–92 on AY267839; Cyp24 (108 bp), 145–163/252–232 onNM_001128915; Cyp27 (78 bp), 157–175/234–214 on NM_000784; and GAPDH (70 bp), 408–430/477–459 on NM_002046. The protocol includes melting for 15 min at 95°C, 40 cycles of three-step PCR including melting for 15 sec at 95°C, annealing for 30 sec at 58°C, elongation for 30 sec at 72°C with an additional detection step of 15 sec at 81°C, followed by a melting curve from 55–95°C at the rate of 0.5°C per 10 sec; annealing was at 55°C and detection was at 76°C. Samples of 25 ng cDNA were analyzed in quadruplicate in parallel with β-actin controls; standard curves (threshold cycle *vs.* log pg cDNA) were generated by log dilutions of from 0.1 pg to 100 ng standard cDNA, and then experimental mRNA starting quantities were calculated from the standard curves and averaged using i-Cycler, iQ software as described previously. The ratios of marker experimental gene to GAPDH mRNA were computed and normalized to control (untreated) samples as 100% [17].

### Down-regulation of VDR Expression in SKBR3 cells by Small Interfering RNA (siRNA)

VDR levels were down-regulated in SKBR3 cells using VDR small inhibitory RNA (siRNA) using standard techniques as before (18). Human VDR gene was targeted by using ON-TARGET plus SMART pool siRNA which consists of four siRNA sequences–siRNA1 5′GCAACCAAGACUACAAGUA3′, siRNA2 5′GCGCAUCAUUGCCAUACUG3′, siRNA3 5′CCAACACACUGCAGACGUA3′, and siRNA4 5′GCAAUGAGAUCUCCUGACU3′ (Dharmacon, Lafayette, CO, Cat# L-003448-00-0005). These pooled siRNAs were used at 100 nM concentrations with standard transfection protocol using lipofectamine 2000 (Invitrogen, Carlsbad, CA). Random siRNA (100 nM) was used as a control. We were able to get ∼75–80% inhibition of VDR protein expression.

### Over-expression of Full-length VDR in SKBR3 cells

VDR was over-expressed in SKBR3 cells by transient transfection using Myc-DDK-tagged human VDR ORF clone (NM_001017535) (Cat# RC219628) obtained from Origene Technologies (Rockville, MD) using standard techniques. Briefly, 2.5×10^5^ cells were plated per well on a 6-well plate 24 hrs prior to transfection. 1.5 µg of control vector or VDR clone was mixed with lipofectamine 2000 (1∶3) and cells were incubated overnight after transfection. Next day, medium was replaced with fresh complete growth medium and cells were allowed to grow for another 24 hrs. VDR protein expression was analyzed by western blot analysis to confirm the transgene expression.

### Immunoblot Analysis of Cells and Patient Tissue Samples

Tumor samples from triple negative (TN) (ER^−^, PR^−^, Her2^−^) and estrogen receptor positive (ER^+^) tumor patients were obtained from the following sources- a) Cooperative Human Tissue Network (CHTN) (http://chtn.nci.nih.gov), (tumor samples were collected using NCI funded resource under OHRP guidelines and waiver of consent (45CFR46.101b) for anonymized samples; b) National Disease Research Interchange (NDRI) (http://ndriresourse.org) (approved biomedical research and IRB protocols from consented breast tumor patients) and c) Breast tumor and Tissue Repository at University of California, Los Angeles (UCLA) (approved biomedical research and IRB protocols from consented breast tumor patients). The research protocol was approved by the Charles Drew University Institutional Review Board (IRB) (permit number: 09-08-2229-03). Protein lysates (30–75 µg) were resolved on 10–12% SDS-PAGE gels, electro-transferred and analyzed for protein expression using the following antibodies- VDR (D-6, sc-13133 Santa Cruz Biotech), RXR-α (D-20, sc-553, Santa Cruz Biotech), CD44 (cat# 3570, Cell Signaling) ERK1/2 (New England Biolab Inc., Ipswich, MA), pERK1/2 (9121L, Cell Signaling), MKP-1 (Sc-8599; Santa Cruz Biotechnology) and GAPDH (Chemicon International Inc., Temecula, CA) and appropriate HRP-linked to secondary antibodies (1∶1000 dilution) (Amersham Corp., Piscataway, NJ). Immunoreactive bands were visualized, scanned and analyzed by Image Quant software [17].

### Analysis of 25D Metabolites

Analysis of 25D metabolites was performed as described previously [19]. Briefly, aliquots of cells were incubated with radiolabeled 25(OH)D substrate (^3^H-25(OH)D, 5 nM) for 5 hrs in serum-free culture medium. The resulting mix of ^3^H-vitamin D metabolites was then extracted from the total cell lipids using initial Sep-pak purification and subsequent HPLC analysis of 1α-hydroxylated and 24-hydroxylated vitamin D metabolites. Direct quantification of ^3^H-25(OH)D, ^3^H-1,25(OH)_2_D and ^3^H-24,25(OH)_2_D utilizing an in-line radiodetector was used to quantify 1α- and 24-hydroxylase activities. Data were reported as fmoles of vitamin D metabolite produced per hr per mg cellular protein.

### Xenotransplants

This study was carried out in strict accordance with the recommendations in the Guide for the Care and Use of Laboratory Animals of the National Institutes of Health. The protocol was approved by the Institutional Animal Care and Use Committee (IACUC) on the Ethics of Animal Experiments of the Charles Drew University of Medicine and Science (permit number: I-1103-261). All surgery was performed under isoflurane anesthesia, and all efforts were made to minimize suffering. Eight week old nude mice were purchased from Harlan Laboratories Inc. (Placentia, CA). Mammary glands of the mice were cleared of endogenous epithelium and humanization of the fat pad was done by injecting irradiated (4 Gy) fibroblasts from human tumors (50,000 cells/100 µl matrigel/fat pad). After two weeks, 10^5^ dissociated cells (suspended in 100 µl matrigel/fat pad) obtained from mammospheres after various treatments were injected into the humanized fat pad of nude mice (12 mice/group) and tumor growth was monitored. Measurements were obtained by caliper length and width measurements at weekly intervals for the duration of the experiment. Tumor volume was calculated as ½ (length×width^2^) [20].

### Statistical Analysis

Data are presented as mean+/− SD, and between group differences were analyzed using ANOVA. If the overall ANOVA revealed significant differences, then pair-wise comparisons between groups were performed by Newman-Keuls multiple group test. All comparisons were two-tailed and p values ≤0.05 were considered statistically significant. The experiments were repeated at least three times, and data from representative experiments are shown.

## Results

### Isolation of Mammospheres with Mammary Cancer Stem Cell Properties from Breast Cancer Cell Lines

**Figure 1:**
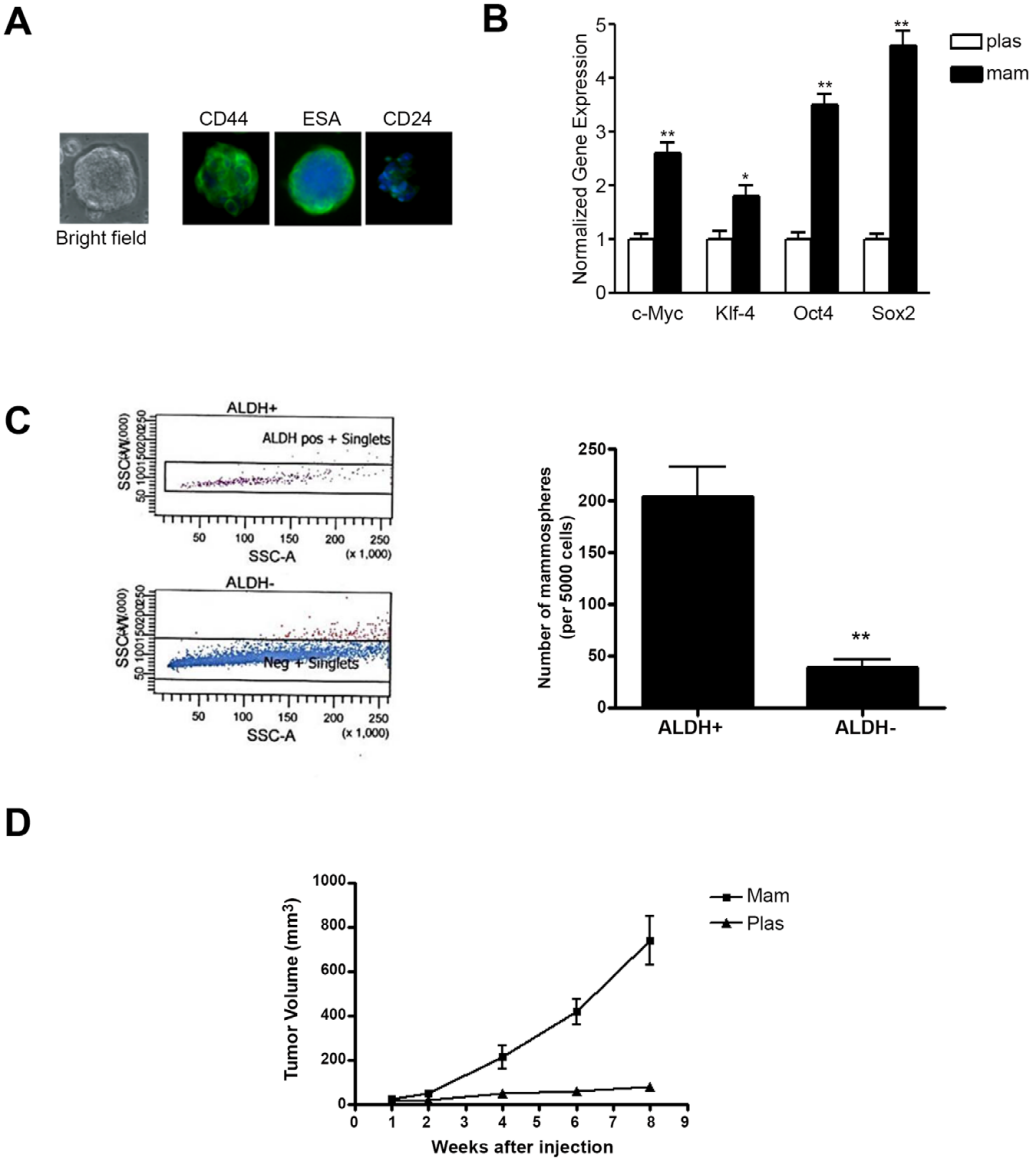
*A*: Left Panel: Bright field photomicrograph of mammospheres isolated from SKBR3 CELLS. Right Panel: Fluorescent micrographs of SKBR3 mammospheres stained with CD44, ESA, and CD24 antigens (green) and DAPI (blue). *B,* Real-time quantitative PCR analysis of c-Myc, Klf4, Oct4, and Sox2 in SKBR3 cells grown under high attachment, plastic (plas) or mammosphere (mam) conditions (*, p≤0.05; **, p≤0.01). *C*, Isolation of ALDH^+^ and ALDH^−^ population from SKBR3 cells and analysis of their mammosphere forming capacity. *D,* Analysis of tumor volume in nude mice by SKBR3 mammospheres and plastic cells. 1×10^5^ cells obtained from dissociated mam or plas were injected into the humanized mammary fat pads of female nude mice and tumor volumes were analyzed at various time points (1–8 weeks).

We examined the mammosphere formation capability of several breast cancer cell lines SKBR3 (Figure 1A, left panel), MCF-7 and HMLE^H-RAS^ and human mammary epithelial (HMLE) cells (data not shown) by culturing them in defined mammosphere media on ultra-low attachment plates. Well-defined heterogeneous populations of mammospheres were obtained from all these cell lines at varying frequencies (1 in every 200–500 cell). Interestingly, SKBR3 cells had the highest frequency of mammosphere formation among various cell lines studied. The self-renewal capability of the mammospheres was examined by repeatedly dissociating the spheres (typically at day 5 of growth) and following the life span by serial passage in mammosphere conditions. Analysis of cell surface antigens showed strong expression of cancer stem cell antigens CD44 and epithelial-specific antigen (ESA) in SKBR3 mammospheres, whereas expression of CD24, epithelial cell marker was low (Fig. 1A, right panel). Expression of key transcription factors Sox2, Oct4, Klf4 and c-Myc, were significantly up-regulated in the mammospheres isolated from SKBR3 breast cancer cells (Fig. 1B). As the ALDH^+^ population has been demonstrated to be enriched in mammary cancer stem cells, we isolated ALDH^+^ and ALDH^−^ cell population by cell sorting (FACS) (Figure 1C, left panel) and analyzed their efficiency to form mammospheres. We found that ALDH^+^ cells had significantly higher mammosphere forming capability (∼one per 25 cells) compared to that of ALDH^−^cells (∼one per 140 cells) (Figure 1C, right panel).

Studies were also carried out to determine the *in vivo* enrichment capacity of stem cell populations in mammospheres isolated from SKBR3 cells. Xenograft formation (5/12) was induced in nude mice by injecting 10^5^ cells from dissociated SKBR3 mammospheres in the humanized mammary fat pads of nude mice for 8 weeks (Figure 1D). When a similar number of more differentiated cells grown on plastic were injected, there was no apparent tumor formation within this time period (Figure 1D).

### Vitamin D Signaling is Selectively Down-regulated in Mammary Cancer Stem Cells

**Figure 2:**
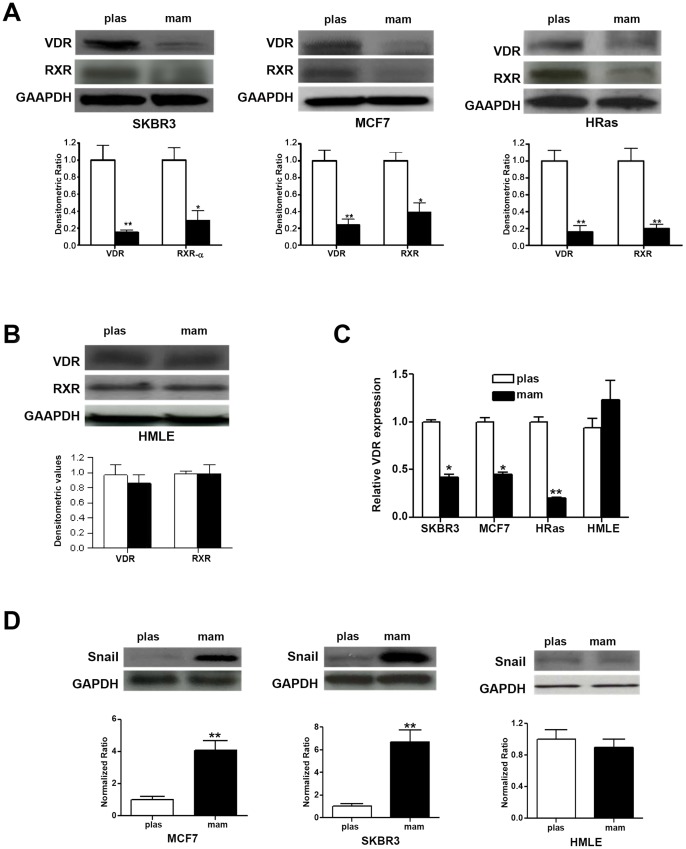
Selective down-regulation of VDR/RXR expression in mammospheres isolated from breast cancer cells *A,* Top panel: 50 µg of total cell lysates isolated from breast cancer cell lines SKBR3 (left), MCF7 (middle) and HRas (right) cells grown under plas or mam conditions and were analyzed for VDR and RXR protein expression by Western blot analysis. Bottom panel: Quantitative densitometric analysis of VDR and RXR expression in SKBR3 (left), MCF7 (middle) and HRas (right) cell normalized to GAPDH (*, p≤0.05; **, p≤0.01). *B,* Top panel: 50 µg of total cell lysates isolated from mammary epithelial HMLE cells grown under plas or mam conditions were analyzed for VDR and RXR protein expression by Western blot analysis. Bottom panel: Quantitative densitometric analysis of VDR and RXR expression normalized to GAPDH (*, p≤0.05; **, p≤0.01). *C,* Real-time quantitative PCR analysis of VDR expression in SKBR3, MCF7 and HRas as well as in HMLE cells grown under plas or mam conditions (*, p≤0.05; **, p≤0.01). *D,* Selective up-regulation of Snail in MCF7 and SKBR3 cells. Top panel; 50 µg of total cell lysates isolated from MCF7 (left panel), SKBR3 (middle panel) or HMLE cells (right panel) grown under plas or mam conditions were analyzed for Snail expression by Western blot analysis. Bottom panel: Quantitative densitometric analysis of Snail expression normalized to GAPDH (**, p≤0.01).

We next examined expression of VDR in breast cancer cells grown as mammospheres or as more differentiated cells grown on high attachment plates. Significantly reduced levels of VDR protein were found in mammospheres isolated from SKBR3 (78.4±7.6%), MCF-7 (68.2±9.6%) and HRas (81.4±9.2%) cells compared to more differentiated cells grown in plastic (Figure 2A). Protein expression of RXR, a nuclear receptor that heterodimerizes with VDR, was also significantly down-regulated in mammospheres from SKBR3 (68.6±11.2%), MCF-7 (58.5±14.4%) as well as HRas (78±6%) cell lines compared to more differentiated cells (Figure 2A). However, there was no significant difference between VDR and RXR protein expression in mammary epithelial HMLE cells propagated either as mammosphere or plastic (Figure 2B). Comparison of gene-expression between plastic and mammosphere cultures also suggests that VDR mRNA was significantly down-regulated in mammospheres isolated from breast cancer cells (MCF7∶56.2±4.6%; SKBR3∶59.5±6.2%; and HRas: 72.4±3.5%) compared to cells grown on plastic; however, there was no significant difference in VDR expression between mammospheres and plastic cultured cells isolated from mammary epithelial HMLE (Figure 2C).

### Selective Induction of Snail in Mammospheres Obtained from Breast Cancer Cells

Expression of the transcription suppressor Snail was assessed in mammospheres and cells grown on high attachment plates from MCF7 and SKBR-3 as well as from HMLE cells. Snail was significantly up-regulated in mammospheres isolated from SKBR3 (6.8±1.2 fold) and MCF7 (4.1±0.3 fold) cells compared to equivalent cells cultured on plastic (Figure 2D, left and middle panel). On the other hand, there was no significant difference in Snail expression between mammosphere and plastic groups in HMLE (Figure 2D, right panel).

### Up-regulation of Epithelial to Mesenchymal Transition (EMT) Gene Signature in Mammospheres

**Figure 3:**
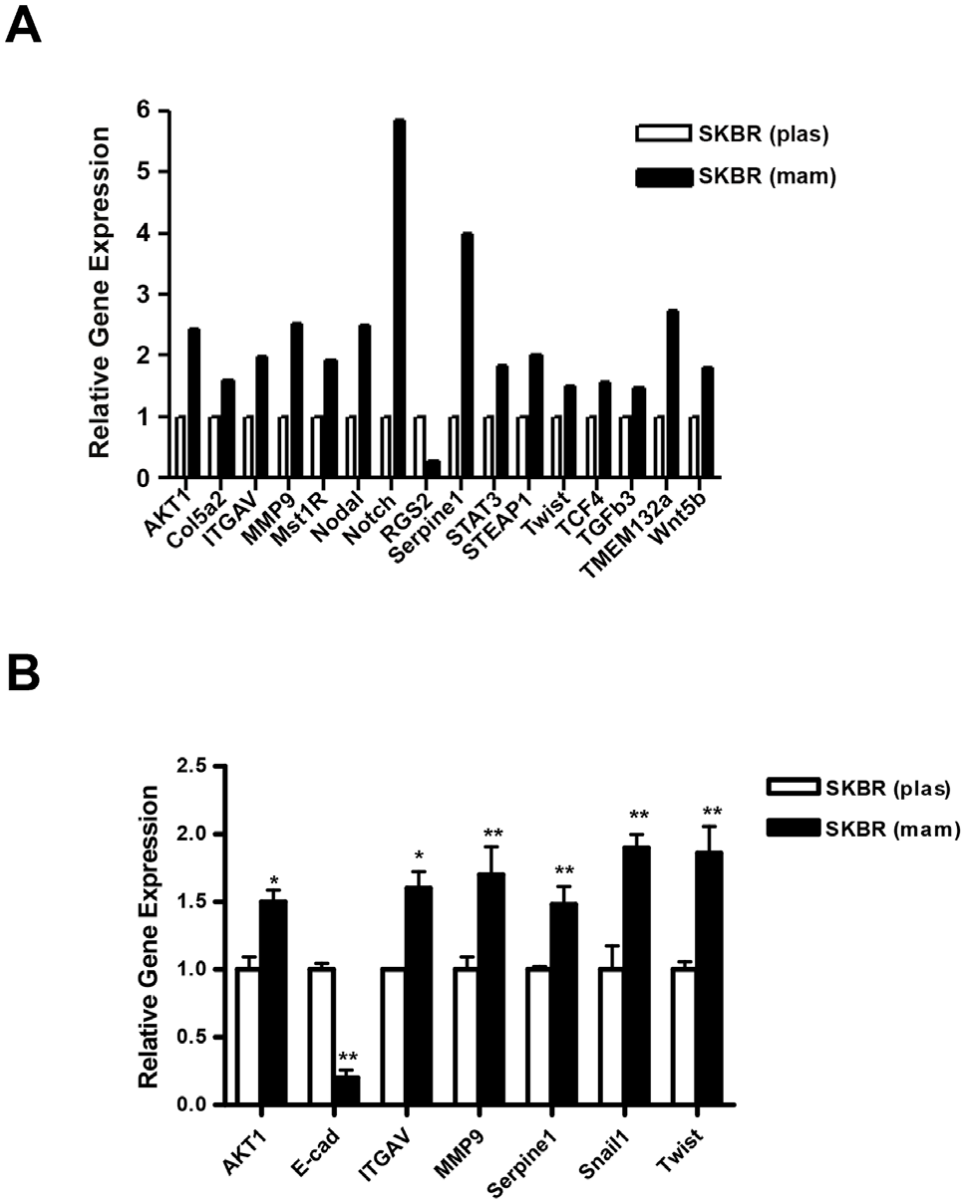
*A,* PCR Array Analysis of EMT-specific genes in SKBR3 cells grown under plas and mams conditions after 4 days. *B,* Validation of PCR Array data by quantitative real time PCR analysis. Experiment was performed in triplicates from three different sets of experiments (*, p≤0.05; **, p≤0.01).

Since Snail, one of the well-known inducers of EMT was found to be up-regulated in mammospheres isolated from breast cancer cells; we compared EMT gene signature in SKBR3 cells grown under plastic and mammospheres conditions. We initially utilized EMT PCR-Array analysis to compare the expression profile of various genes involved during EMT transition between plastic and mammospheres groups (Figure 3A). We found up-regulation of Akt1 (2.44 fold); collagen, type 5a2 (Col5a2) (1.60 fold); integrin alpha 5 (ITGAV) (1.99 fold); MMP9 (2.53 fold); Serpine 1 (3.98 fold); six-transmembrane epithelial antigen of prostate 1 (STEAP1) (2.01 fold), TCF4 (1.56 fold); twist (1.50 fold); transmembrane protein 132a (TMEM132a) (2.73 fold) and Wnt 5a (1.81 fold) and down-regulation of regulator of G-protein signaling 2 (RGS2) (62%) among several other genes in mammospheres compared to the plastic group (Figure 3A). Also, we found up-regulation of macrophage stimulating 1 receptor (Mst1R) (1.92 fold); Nodal (2.5 fold) as well as Stat 3 (1.82 fold) genes in mammospheres, which are highly implicated in migration and motility of cells (Figure 3A). Next, we performed quantitative real-time PCR analysis to confirm and validate the changes in EMT gene signature from plastic and mammospheres groups (Figure 3B). We found a significant increase in Akt1 (1.5±0.15 fold); ITGAV (1.6±0.21 fold); MMP9 (1.7±0.26 fold); Serpine 1 (1.48±0.23 fold); Snail (1.92±0.17 fold) and Twist (1.86±0.24 fold) as well as a significant decrease in E-cad (80.2±8%) gene expression in mammospheres compared to the plastic group (Figure 3B). Our data, therefore, suggest that our initial observation of down-regulation of VDR and concurrent induction of Snail expression in mammospheres is associated with significant changes in gene expression profiles that promote EMT.

### Effect of Genetic Manipulation of VDR Expression on Key EMT Regulators and the Mammosphere Forming Capability in Breast Cancer Cells

**Figure 4:**
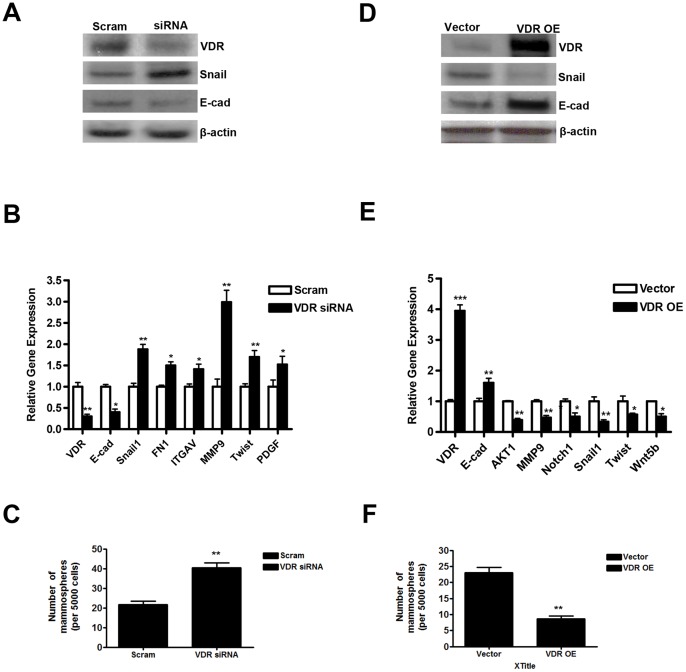
*A,* Inhibition of VDR expression in SKBR3 cells by siRNA and analysis of VDR, Snail and E-cad protein expression. SKBR3 cells were transfected either with scrambled siRNA (Scram) or On-target smart pool VDR siRNA (siRNA) using standard techniques and protein expression was analyzed by western blot analysis. *B,* Analysis of EMT-specific gene signature in Scam and VDR siRNA transfected cells (*, p≤0.05; **, p≤0.01). *C,* Analysis of mammospheres forming capability in Scam and VDR siRNA transfected cells. 5,000 cells after transfection were plated on 24 well ultra-low attachment plates and total numbers of mammospheres formed were analyzed (**, p≤0.01). *D,* Over-expression of full length human VDR gene and analysis of VDR, Snail and E-cad protein expression by western blot. *E,* Analysis of EMT-specific gene signature in cells transfected either with control vector or full-length human VDR (*, p≤0.05; **, p≤0.01). *F,* Analysis of mammospheres forming capability in cells transfected either with control vector or VDR over-expressing (VDR OE) plasmid. 5,000 cells after transfection were plated on 24 well ultra-low attachment plates and total numbers of mammospheres formed were analyzed (**, p≤0.01).

To further explore the functional role of VDR in modulating key proteins and genes involved during EMT transition and correlate their possible association with the ability to form mammospheres, we performed both VDR knock out (Figure 4A–C) and VDR over-expression (OE) (Figure 4 D-F) experiments in SKBR3 cells. Using VDR on-target plus pool of siRNAs directed against human VDR, we were able to knock down ∼75% expression of VDR. Inhibition of VDR expression by siRNA pools resulted in appreciable up-regulation of Snail but down-regulation of E-cadherin (E-cad) proteins compared to the scrambled RNA treated group (Figure 4A). We next, analyzed the gene expression profile of key EMT markers from mammospheres isolated from scrambled (Scram) as well as from VDR siRNA transfected cells. We found a significant decrease in VDR (70.7±9%) and E-cad (60.0±13%) genes but significant up-regulation of Snail1 (1.88±0.2 fold); fibronectin 1 (FN1) (1.5±0.15 fold); ITGAV (1.41±0.21 fold); MMP9 (2.99±0.31 fold); Twist (1.70±0.12 fold) as well as PDGF (1.52±0.27 fold) (Figure 4B). We next compared mammosphere forming abilities of the cells treated either with Scam or VDR siRNA. We found a significant increase in the mammosphere forming ability of cells treated with VDR siRNA (1.93±0.27 fold) compared to the Scram RNA treated group (Figure 4C).

We also performed VDR over-expression in SKBR3 cells using human VDR ORF clone purchased from Origene. We were able to significantly up-regulate VDR protein expression (4–5 fold) in VDR OE group. This up-regulation of VDR protein expression was associated with down-regulation of Snail but up-regulation of E-cad protein (Figure 4D). Analyses of gene expression profile of select EMT pathway genes between mammospheres isolated from control vector and VDR OE groups demonstrate a significant up-regulation of VDR (3.98±0.34 fold) and E-cad (1.6±0.26 fold) gene expression but significant down-regulation of Akt1 (61.0±9%); MMP9 (53.2±12%); Notch1 (50.0±21%); Snail1 (66.5±11%); Twist (43.0±8%) and Wnt5b (51.4±17%) (Figure 4E). Also, VDR OE cells had significantly decreased (2.84±48 fold) ability to form mammospheres (Figure 4F). Therefore, our combined data from VDR knock out as well as VDR OE experiments in SKBR3 cells clearly suggest that manipulation of VDR expression dictates the ability of cancer cells to form mammospheres by regulation of Snail/E-cad-mediated pathway, which is essential during EMT process.

### Growth Factor Independence of VDR Down-regulation in Mammospheres

**Figure 5:**
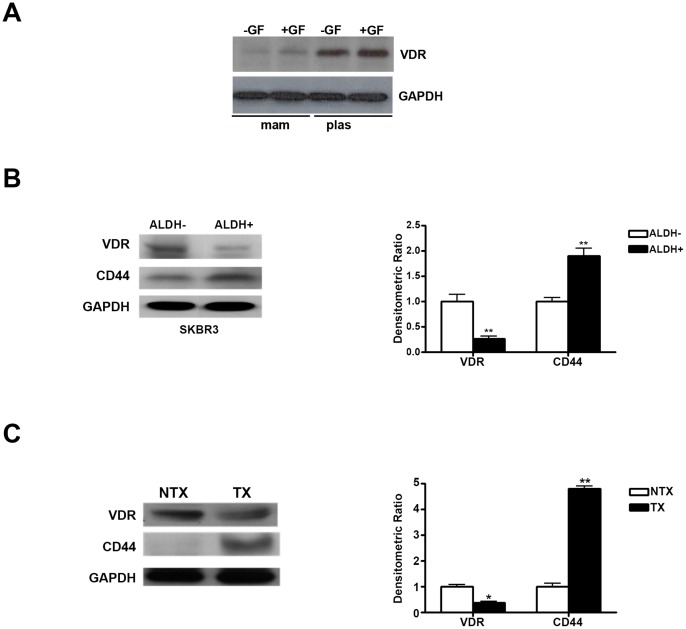
*A,* Analysis of VDR expression in SKBR3 cells grown under mam or plas conditions with or without growth factors (GF: EGF plus FGF). 30 µg of cell lysates were electrophoretically separated and analyzed by western blot analysis using anti-VDR antibody. Membranes were stripped and re-probed using anti-GAPDH antibody for loading controls. *B (Left Panel),* Analysis of VDR and CD44 protein expression in ALDH^−^ and ALDH^+^ populations isolated from SKBR3 cells. 30 µg of cell lysates obtained from ALDH^+^ and ALDH^−^ cells were electrophoretically separated and analyzed by western blot analysis using anti-VDR or anti-CD44 antibodies. Membranes were subsequently stripped and re-probed with anti-GAPDH antibody for loading controls. *B* (*Right Panel)*, Normalized densitometric ratios showing relative expression levels of VDR and CD44. *C (Left Panel),* Analysis of VDR and CD44 protein expression in non-transplantable xenografts (NTX) and transplantable xenografts (TX). 30 µg of total cell lysates obtained from non-transplantable xenografts (NTX) and transplantable xenografts (TX) were electrophoretically separated and analyzed by western blot analysis using anti-VDR or anti-CD44 antibodies. Membranes were stripped and re-probed with anti-GAPDH antibody for loading controls (*, p≤0.05; **, p≤0.01). *C (Right Panel),* Normalized densitometric ratios showing relative expression levels of VDR and CD44.

Expression of VDR protein was assessed in SKBR3 cells grown under mammosphere and plastic conditions either in presence or absence of growth factors (GF) that included a combination of EGF (10 ng/ml) and FGF (10 ng/ml). Under both conditions, the level of VDR expression was not significantly different (Figure 5A) within either group, suggesting that the down-regulation of VDR mRNA and protein in mammosphere is independent of incubation with the above doses of EGF and FGF. However, the level of VDR expression in mammosphere group was much lower compared to the plastic group as expected (Figure 5A).

### Inverse Relationship between VDR and CD44 Expression in ALDH^+^ cells and Transplantable Xenografts

To further validate our initial findings that mammospheres enriched in MCSCs express lower levels of VDR expression, we isolated ALDH^+^ and ALDH^−^ populations from SKBR3 cells by cell sorting and compared the levels of VDR and CD44 expression between the groups. We found that ALDH^+^ population had significantly lower (72.6±5.4%; p≤0.01) levels of VDR expression compared to the ALDH^−^ populations. On the other hand, CD44 was significantly up-regulated (88±9%; p≤0.01) in ALDH^+^ population (Figure 5B). We next compared the expression levels of VDR and CD44 between a highly aggressive and transplantable xenograft line (TX) obtained after injection of HRas cells in the nude mice to that of a non-transplantable xenograft line (NTX). VDR expression was significantly down-regulated (62.4±6.3%, p≤0.05), whilst CD44 protein expression was significantly up-regulated (4.8±0.44 fold, p≤0.001) in TX compared to the NTX (Figure 5C).

### Selective Down-regulation of VDR Expression in Aggressive TN Tumors Compared to the ER+ Tumors and it’s Association with Increased Expression of CD44

**Figure 6:**
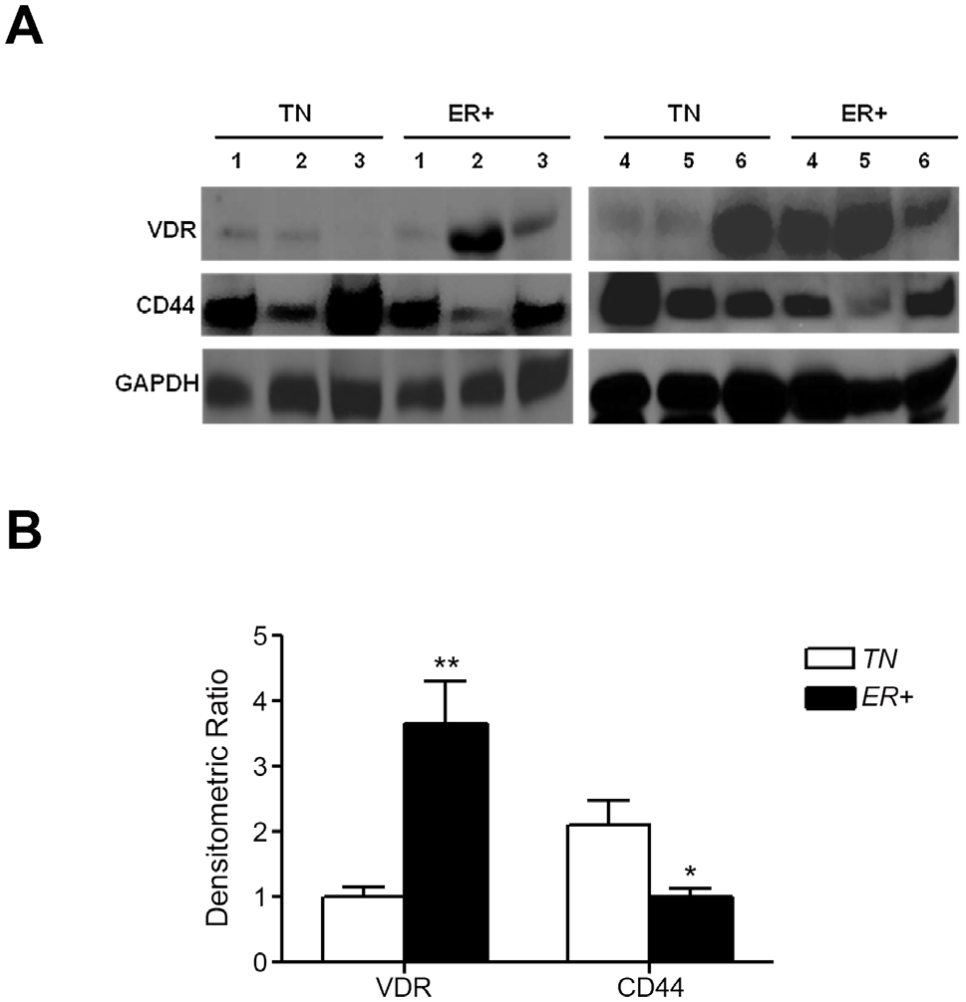
*A,* Analysis of VDR and CD44 protein expression in tumor biopsies from triple negative (TN) and estrogen receptor positive (ER^+^) breast cancer patients. 50 µg lysates from 6 tumor samples each from TN and ER^+^ were analyzed for VDR and CD44 protein expression by Western blot analysis. *B,* Normalized densitometric values for VDR and CD44 expression from TN and ER^+^ tumor patients (*, p≤0.05; **, p≤0.01).

To test whether VDR levels are down-regulated in more aggressive tumors, VDR and CD44 expression was assessed in fresh human tumor samples obtained from aggressive triple negative (TN) and estrogen receptor positive (ER^+^) tumors (Table 1). Western blot analyses of tumor specimens (6 TN and 6 ER^+^) suggested an inverse relationship between tumor aggressiveness and VDR expression (Figure 6A–B). TN breast tumors expressed significantly lower VDR (76.4±6.2%) and higher CD44 (105.6±8.7%) expression compared to ER^+^ tumor groups (Figure 6A–B).

**Table 1:** Patient and tumor sample characteristics.

Tumor/Source	Ethnicity	Age (Yrs)	Lymph nodestatus	PathologicalStage	Histological Grade	TumorSize(cm)
TN1/CHTN	white	48	negative	IIB	Infiltrating mammary carcinoma	6.2
TN2/CHTN	white	78	positive	IA	Infiltrating mammary carcinoma	5.3
TN3/CHTN	white	86	negative	IIA	Infiltrating mammary carcinoma	3
TN4/CHTN	white	75	positive	IIIA	Invasive ductal carcinoma	5.7
TN5/NDRI	white	46	negative	IIA	Invasive ductal carcinoma	2.5
TN6/NDRI	white	68	positive	IA	Invasive ductal carcinoma	4.8
ER+1/UCLA	white	48	negative	IIA	Infiltrating ductal carcinoma	4.8
ER+2/UCLA	white	58	positive	IIB	Infiltrating lobular carcinoma	4.5
ER+3/UCLA	white	51	positive	IIIC	Infiltrating ductal carcinoma	4.2
ER+4/CHTN	white	59	positive	IIIA	Infiltrating ductal adenocarcinoma	6.2
ER+5/CHTN	white	57	positive	IV	Invasive mammary carcinoma(multifocal)	7.3
ER+6/CHTN	white	59	positive	IIIA	Infiltrating mammary carcinoma	3.5

Triple negative (TN) and estrogen receptor positive tumor (ER^+^) human breast cancer tissues were obtained from various sources. CHTN, Co-operative Tissue Network, Breast tumor and tissue repository at UCLA (UCLA) and National Drug Research Interchange (NDRI).

### Mammospheres are Resistant to Vitamin D Treatment

**Figure 7:**
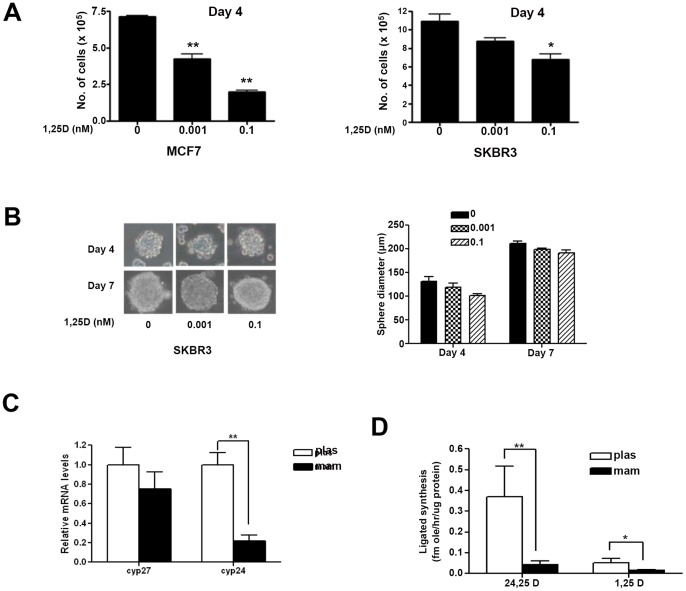
*A,* Effect of 1,25D treatment on MCF7 (left) and SKBR3 (right) cell proliferation. Cells were treated with 1,25D (0–0.1 nM) and allowed to proliferate for 4 days under high attachment conditions and cell numbers were counted by trypan blue method (*, p≤0.05; **, p≤0.01). Medium was replaced after every 48 hrs with appropriate concentrations of 1,25D. *B,* SKBR3 (2×10^4^) cells were plated under mammosphere conditions on a 12-well ultra-low attachment plates in presence of different concentrations of 1,25D (0–0.1 nM) and allowed to grow under mammosphere conditions for 4 or 7 days and sphere diameters were measured. Appropriate concentrations of 1,25D were additionally supplemented in the culture medium after every 48 hours. Left Panel: Micrographs were taken at 100×magnification. Right Panel: Quantitative analysis of average diameter computed from 20 different fields from each treatment group. *C,* Quantitative real-time PCR analysis of Cyp27 B1 and Cyp24A1 mRNA expression from MCF-7 cells grown under plas or mam conditions after 4 days of plating (**, p≤0.01). *D,* HPLC analysis of 24,25D3 and 1,25D synthesis in cells grown under plastic or mammos conditions from MCF-7 cells after 4 days of plating (*, p≤0.05; **, p≤0.01).

Breast cancer cells cultured in various concentrations of 1,25D under high attachment conditions were highly sensitive to 1,25D, which inhibited cell proliferation in both MCF7 (58±3% at 0.001 nM and 76±5% at 0.1 nM) and SKBR3 (34.5±6% at 0.1 nM) cells (Figure 7A). However, the proliferation of the mammospheres as measured by their average diameter, did not change significantly either at 4 or 7 days after treatment with 1,25D (0–100 nM) (Figure 7B). There was no significant difference in expression of mRNA for the vitamin D-activating enzyme CYP27B1 between the groups; however, there was a significant decrease in expression of the vitamin D-catabolic enzyme CYP24A1 (79±6%) in mammospheres (Figure 7C). Synthesis of 24,25D (CYP24A1 activity) and 1,25D (CYP27B1 activity) from precursor 25D in cells grown under both plastic and mammospheres conditions for 4 days was studied by HPLC analysis (Figure 7D). There was a significant decrease in both CYP24A1 and CYP27B1 activities in mammosphere vs. plastic cultures (Figure 7D).

### Nitric Oxide Induced MKP-1 Expression and pERK1/2 Dephosphorylation in Mammospheres Isolated from SKBR3 cells and Potentiated the Growth Inhibitory Effects of 1,25D both In Vitro and In Vivo

**Figure 8:**
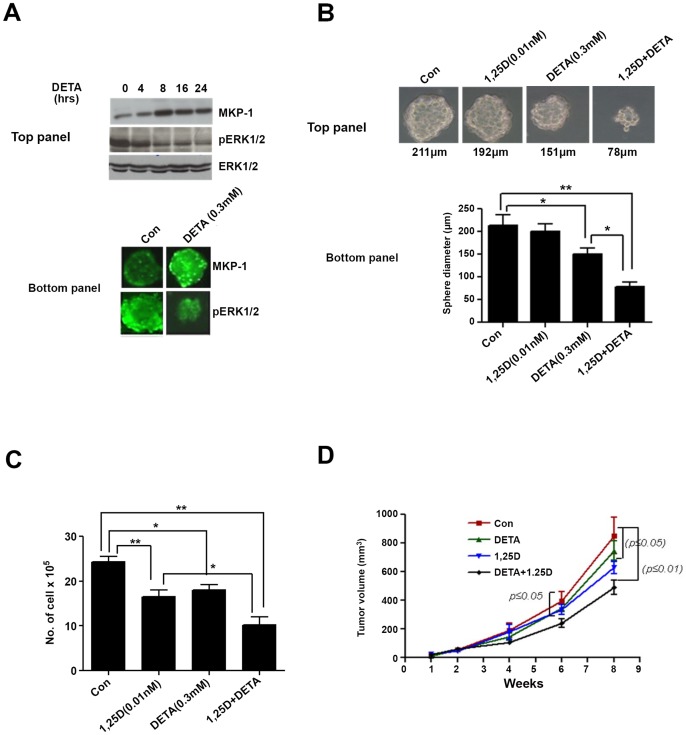
*A,* Top panel: MKP-1 induction and dephosphorylation of pERK1/2 in DETA NONOate (DETA) treated HRas mammospheres. Bottom Panel: Immunofluorescence analysis of MKP-1 and pERK1/2 in control and DETA (0.3 mM) treated mammospheres after 24 hrs. *B,* Top panel: Photomicrographs of HRas cells plated under mammosphere conditions and allowed to grow in medium containing either 1,25D (0.1 nM) and DETA (0.3 mM) alone or in combination for 5 days. Bottom panel; Average diameter of mammospheres computed from 20 different fields from each treatment groups (*, p≤0.05; **, p≤0.01). *C,* 5×10^5^ HRas cells were allowed to seed on T-25 flasks and treated with 1,25D and DETA either alone or in combination. Total number of cells were counted after 5 days (*, p≤0.05; **, p≤0.01). *D,* HRas cells were plated under mammospheres conditions and treated with DETA (0.3 mM), or 1,25D (0.1 nM) either alone or in combination for 3 days. Mammospheres were dissociated and 1×10^5^ cells from each treatment groups were injected into nude mice and tumor volumes were analyzed at various time points (1–8 weeks) (*, p≤0.05; **, p≤0.01 compared to the Con group).

Histone deacetylase (HDAC) inhibitors are reported to augment the growth inhibitory effects of 1,25D in a TN breast cancer cell line, MDA-MB-231 by releasing nitric oxide (NO) [21]. We have previously demonstrated that NO treatment of breast cancer cells leads to the induction of MKP-1 followed by dephosphorylation of ERK1/2, which was essential for inducing apoptosis in breast cancer cells [22]. More recently, we also found that stable nuclear pERK1/2 was present at basal level in these mammospheres (Pervin et. al, under review). Accordingly, we tested whether a combination of NO and 1,25D would be more effective in inhibiting the proliferation of mammospheres *in vitro* and tumor size in nude mice *in vivo*. DETA NONOate treatment led to the induction of MKP-1 protein expression in a time-dependent manner, and this effect was associated with a concomitant decrease in pERK1/2 expression in mammospheres (Figure 8A, top panel and bottom panel). Cells treated either with 1,25D (0.01 nM) and DETA NONOate alone or in combination were grown under mammosphere conditions for 4 days after which the sphere diameters were measured. 1,25D treatment alone did not significantly change the average size of mammospheres. However, in combination with DETA NONOate, it significantly decreased the mammosphere size (63.4±18.1%) (Figure 8B). A significant decrease in mammosphere size (30.1±8.4%) was also observed with DETA NONOate treatment alone (Figure 8B). By contrast, when cells were allowed to grow in plastic either in the presence of 1,25D (32.04±3.7%), DETA NONOate (25.8±2.4%) alone or in combination (58.3±4.4%), significant inhibition in cell proliferation of HRas cells was observed after 4 days (Figure 8C). When 1×10^5^ cells obtained from dissociated mammospheres after 4 day treatments with DETA NONOate and 1,25D either alone or in combination were injected into nude mice, there was a significant inhibition of tumor volume compared to the control group after 6 weeks (con vs. DETA+1,25D: 39.2±8.2%) and 8 weeks (con vs. 1,25D: 26.3±4.4%; con vs. DETA+1,25 D: 43.4±6.3%) (Figure 8D). On the other hand, there was only 12.3±2.2% inhibition in tumor volume in DETA (NO-donor) treated mammosphere group compared to the control group after 8 weeks of injection (Figure 8D).

## Discussion

We show that mammospheres enriched in ALDH+ cells from breast cancer cell lines express a clinically relevant ESA^high^, CD44^high^, and CD24^low^ cell phenotype that can be passaged multiple times and that can form transplantable xenografts in nude mice. An important finding of this study was the down-regulation of VDR/RXR in the mammospheres from breast cancer cell lines when compared to differentiated cells grown on high attachment plates. This effect was independent of growth factors present in the mammosphere medium. Although there are reports that VDR levels are low in aggressive TN breast cancer cell lines, to our knowledge, this is the first report demonstrating the suppression of this receptor in mammospheres. It has been reported that VDR deletion results in a marked accumulation of hematopoietic stem cells in the spleen that can be reversed by calcium dietary supplementation [23].

Further, our studies showed that VDR expression was suppressed while CD44 was up-regulated in transplantable xenografts (TX), ALDH^+^ population enriched in MCSCs as well as in aggressive breast tumors. In breast carcinomas, CD44 has been identified as a key cell-surface marker for various cancer stem cells. CD44 expression in human breast cancer cells enhances self-renewal, mammosphere growth as well as drug resistance [24]–[27]. The ability of vitamin D to suppress CD44 expression in breast cancer cells may suggest that manipulation of vitamin D levels may reduce tumor burden. Our findings of down-regulation of VDR expression in aggressive TN tumors compared to ER+ tumors has limitations as the data is derived only from a limited set of tumor biopsies and therefore, should be interpreted carefully. Another indication that vitamin D regulates stem cells comes from a study where p63, which regulates the proliferative potential of epithelial stem cells, was found to specifically bind to VDR promoter and transcriptionally induce the receptor [28]. Unlike MCSCs and aggressive breast tumors, high levels of functional VDR have been found in normal mammary glands mediating the effects of 1,25D on growth and differentiation of these cells [29]–[30]. Increased numbers of undifferentiated mammary gland terminal end buds have been reported in VDR null mouse, suggesting that vitamin D promotes differentiation during early mammary gland development [30]. In our study, we observed that expression of VDR in the mammary epithelial cells grown on high attachment plastic plates as well as under mammosphere conditions were comparable. Activities of the enzymes that degrade (CYP24A1) and activate vitamin D (CYP27B1) were suppressed in MCSCs, but not in cancer cells propagated under high attachment conditions. This result contradicts other reports where CYP24A1 expression has been shown to be augmented in carcinomas compared to benign lesions [31]. The reason for these differences is not clear.

Mammospheres isolated from breast cancer cell lines were resistant to 1,25D treatment, which contrasts its effects on cancer cells grown under high attachment conditions. It has been demonstrated that MCSCs are not only resistant to various therapies, but that chemotherapy increases the number of stem cells in residual tumors [12]. However, a number of dietary compounds have been found to directly or indirectly affect cancer stem cell self-renewal pathways including pancreatic, breast cancer and melanoma [32]–[33]. High levels of circulating 25D as found in epidemiological studies, have been associated with reduced risk of ovarian and pancreatic cancers [34]–[35]. Accordingly, it may be important to combine vitamin D with other compounds to increase therapeutic efficacy. Since high concentrations of active 1,25D alone produced only marginal effects on mammosphere proliferation, it will be important to synergize this therapeutic molecule with proven therapeutic agents.

Our data provides a direct effect of manipulation of VDR expression either by siRNA-mediated knock down of VDR or over-expression of full length human VDR in mammospheres on gene and protein expression of Snail (mesenchymal) and E-cad (epithelial), two key regulators of EMT pathway. The Snail family of transcription factors are known to suppress VDR expression and are key regulators of epithelial to mesenchymal transition (EMT), a process in which epithelial cells lose their apical and basolateral polarity and degrade basement membrane extracellular matrix components during tumor invasion [15], [36]–[37]. Loss of VDR expression during colon cancer progression is related to Snail up-regulation, suggesting that high levels of Snail may be responsible for the failure of therapy with vitamin D analogues in patients [38]. Disruption of VDR-mediated signaling has been reported for several neoplastic cell lines, and is not only restricted to Snail-mediated mechanisms. Deregulation of VDR-associated co-activators and repressors has been associated with impaired sensitivity to 1,25D in a variety of breast and prostate cancer cell lines [39].

Expression of Snail was up-regulated in mammospheres from breast cancer cells, but was lower in cells on high attachment plates. Inhibition of VDR expression was associated with significant decrease in E-cad (epithelial marker) but a simultaneous increase in Snail1 and Twist among other mesenchymal markers. The mammosphere forming capability of cells were found to be significantly increased after siRNA-mediated inhibition of VDR expression in SKBR3 cells. Similarly, over-expression of VDR significantly reduced the ability of these cells to form mammospheres, an effect which was associated with inhibition of Snail 1 but induction of E-cad. Our data presented here clearly suggest that elevated expression of Snail may be one of the underlying mechanisms for decreased VDR expression and CYP24A1 activity observed in mammospheres.

Various strategies have been proposed to counteract aberrant responses to 1,25D in neoplastic cells. For example, VDR signaling is known to be epigenetically modified with histone deacetylase (HDAC) inhibitors, leading to enhanced anti-proliferative effects of 1,25D [38]. As HDAC inhibitors are reported to release NO [21], the findings that 1,25D in combination with HDAC inhibitors resulted in re-expression of anti-proliferative target genes in TN breast cancer cell line MDA-MB-231 [40] support our findings that combinatorial therapy using 1,25D and low physiological dose of NO may reduce the proliferative ability of MCSCs and thereby inhibit tumor initiation and tumor mass. We have previously reported that aggressive breast cancer cell lines are highly sensitive to NO- mediated cytostasis and apoptosis [41]. NO induces MKP-1, which dephosphorylates ERK1/2 to facilitate Bax integration in to the mitochondrial membrane and increase cytochrome c release from the mitochondria [22], [42]. There are reports that constitutive activation of ERK1/2-MAPK signaling pathway impairs vitamin D signaling in human prostate epithelial cells [43]. In this study, we found only high concentrations of NO donor, DETA-NONOate, were able to reduce the proliferation of mammospheres from breast cancer cells. However, by demonstrating similar results with a combination of low concentration of NO and 1,25D our data provide an alternative strategy for correcting corrupted VDR signaling in breast cancer cells, at least in the context of MCSCs. In future studies it will be interesting to explore the broader translational potential of various NO-donors in combination with vitamin D metabolites and analogs for the treatment of aggressive breast tumors.

In summary, data presented here show for the first time that VDR-mediated signaling in MCSCs enriched mammospheres play a key role in dictating their overall oncogenic program by regulating Snail/E-cad/EMT pathway. Mammospheres exhibit relative insensitivity to the anti-proliferative effects of vitamin D and abrogation of this effect using combinatorial therapies may provide new translational strategies for the use of vitamin D in treating breast cancer. Combinations of 1,25D or its synthetic analogs with other anti-cancer agents have demonstrated synergistic interactions in a variety of studies 44–45. However, several concerns related to the hypercalcemic effects associated with very high doses of 1,25D used to treat breast tumor patients exist. Dexamethasone is reported to significantly improve 1,25D anti-tumor efficacy *in vitro* and *in vivo* through direct effects on VDR [46]. Combination of 1,25D with aromatase inhibitors has been shown to enhance anti-proliferative effects on breast cancer cells [47]–[48]. The expression and function of aromatase and other estrogenic enzymes in MCSCs and their potential modulation by vitamin D metabolites is yet unclear but is likely to provide a fruitful avenue for future studies. Use of MCSCs *in vitro* and *in vivo* provides invaluable model system to explore the molecular mechanisms and efficacy of combinatorial therapies and may provide new translational strategies for the use of vitamin D.
