# Correction: Down-Regulation of Vitamin D Receptor in Mammospheres: Implications for Vitamin D Resistance in Breast Cancer and Potential for Combination Therapy

**DOI:** 10.1371/annotation/5326d117-3f31-4e43-a5c4-9e1fb41719e9

**Published:** 2013-10-21

**Authors:** Shehla Pervin, Martin Hewison, Melissa Braga, Lac Tran, Rene Chun, Amer Karam, Gautam Chaudhuri, Keith Norris, Rajan Singh

In the Mammospheres are Resistant to Vitamin D Treatment section within the Results Section all instances of nM should appear as μM. One exception in the last sentence; the value (0-100 nM) is correct.

There are errors in Figures 1, 7, and 8. Please see the corrected Figures here:

Figure 1: 

**Figure:**
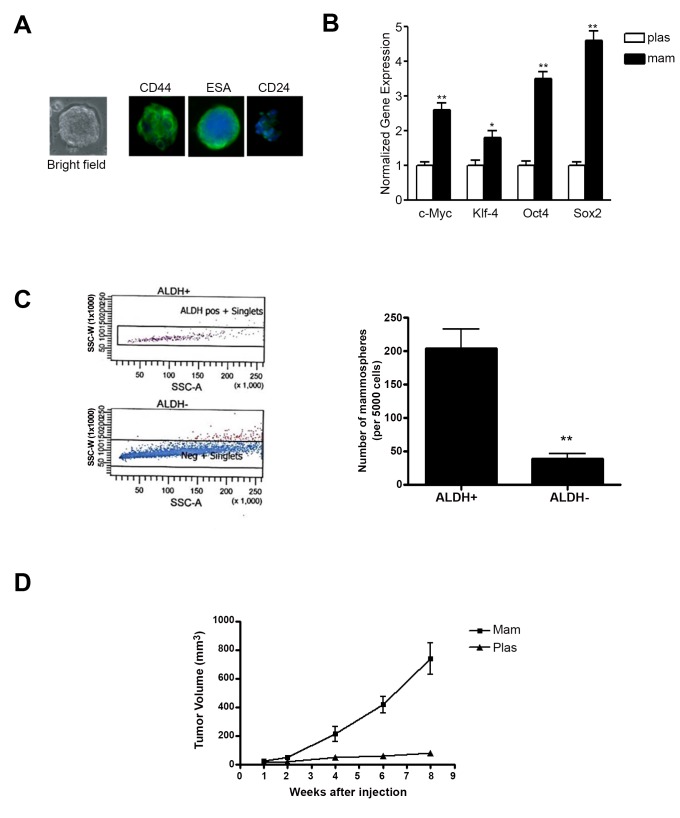


Figure 7: 

**Figure:**
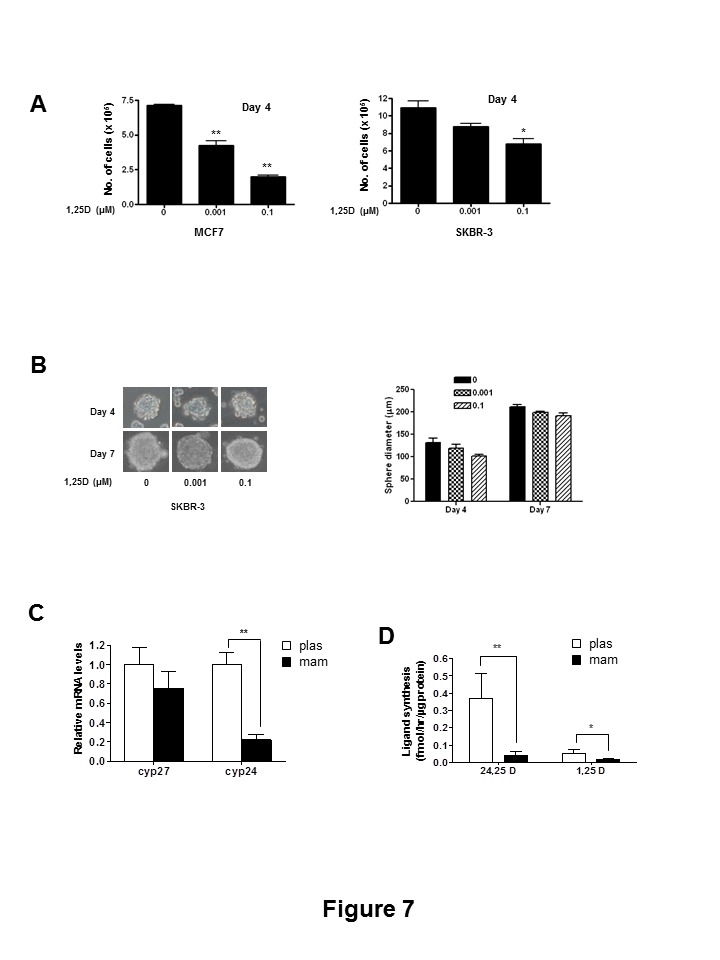


Figure 8: 

**Figure:**
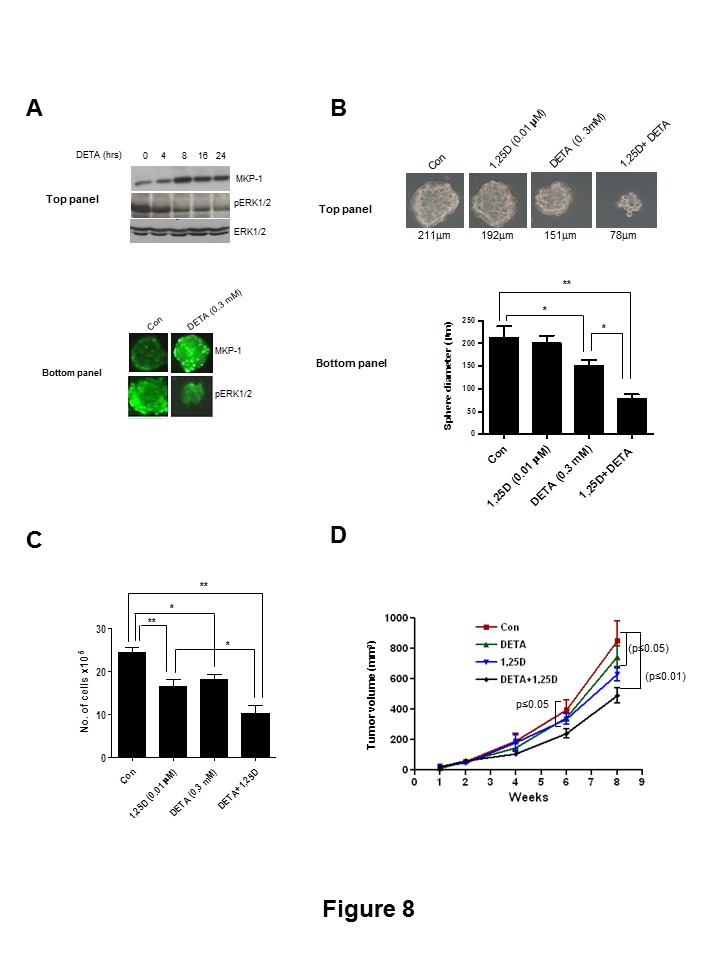


The corrected figure legend of Figure 7 is:

Cells were treated with 1,25D (0–0.1 μM) and allowed to proliferate for 4 days under high attachment conditions and cell numbers were counted by trypan blue method (*, p≤0.05; **, p≤0.01). Medium was replaced after every 48 hrs with appropriate concentrations of 1,25D. B, SKBR3 (2×104) cells were plated under mammosphere conditions on a 12-well ultra-low attachment plates in presence of different concentrations of 1,25D (0–0.1 μM) and allowed to grow under mammosphere conditions for 4 or 7 days and sphere diameters were measured. Appropriate concentrations of 1,25D were additionally supplemented in the culture medium after every 48 hours. Left Panel: Micrographs were taken at 100×magnification. Right Panel: Quantitative analysis of average diameter computed from 20 different fields from each treatment group. C, Quantitative real-time PCR analysis of Cyp27 B1 and Cyp24A1 mRNA expression from MCF-7 cells grown under plas or mam conditions after 4 days of plating (**, p≤0.01). D, HPLC analysis of 24,25D3 and 1,25D synthesis in cells grown under plastic or mammos conditions from MCF-7 cells after 4 days of plating (*, p≤0.05; **, p≤0.01).

doi:10.1371/journal.pone.0053287.g007

The corrected figure legend of Figure 8 is:

Bottom Panel: Immunofluorescence analysis of MKP-1 and pERK1/2 in control and DETA (0.3 mM) treated mammospheres after 24 hrs. B, Top panel: Photomicrographs of HRas cells plated under mammosphere conditions and allowed to grow in medium containing either 1,25D (0.1 μM) and DETA (0.3 mM) alone or in combination for 5 days. Bottom panel; Average diameter of mammospheres computed from 20 different fields from each treatment groups (*, p≤0.05; **, p≤0.01). C, 5×105 HRas cells were allowed to seed on T-25 flasks and treated with 1,25D and DETA either alone or in combination. Total number of cells were counted after 5 days (*, p≤0.05; **, p≤0.01). D, HRas cells were plated under mammospheres conditions and treated with DETA (0.3 mM), or 1,25D (0.1 μM) either alone or in combination for 3 days. Mammospheres were dissociated and 1×105 cells from each treatment groups were injected into nude mice and tumor volumes were analyzed at various time points (1–8 weeks) (*, p≤0.05; **, p≤0.01 compared to the Con group).

doi:10.1371/journal.pone.0053287.g008 

